# Supplementary material for: Interferon signalling and non-canonical inflammasome activation promote host protection against multidrug-resistant Acinetobacter baumannii
Source: Commun Biol. 2024 Nov 12;7:1494. doi: 10.1038/s42003-024-07204-3 (PMC11557958; doi:10.1038/s42003-024-07204-3)
Supplement: Supplementary file 2 — Supplementary Information [file 42003_2024_7204_MOESM2_ESM.pdf]

Supp Figure 1

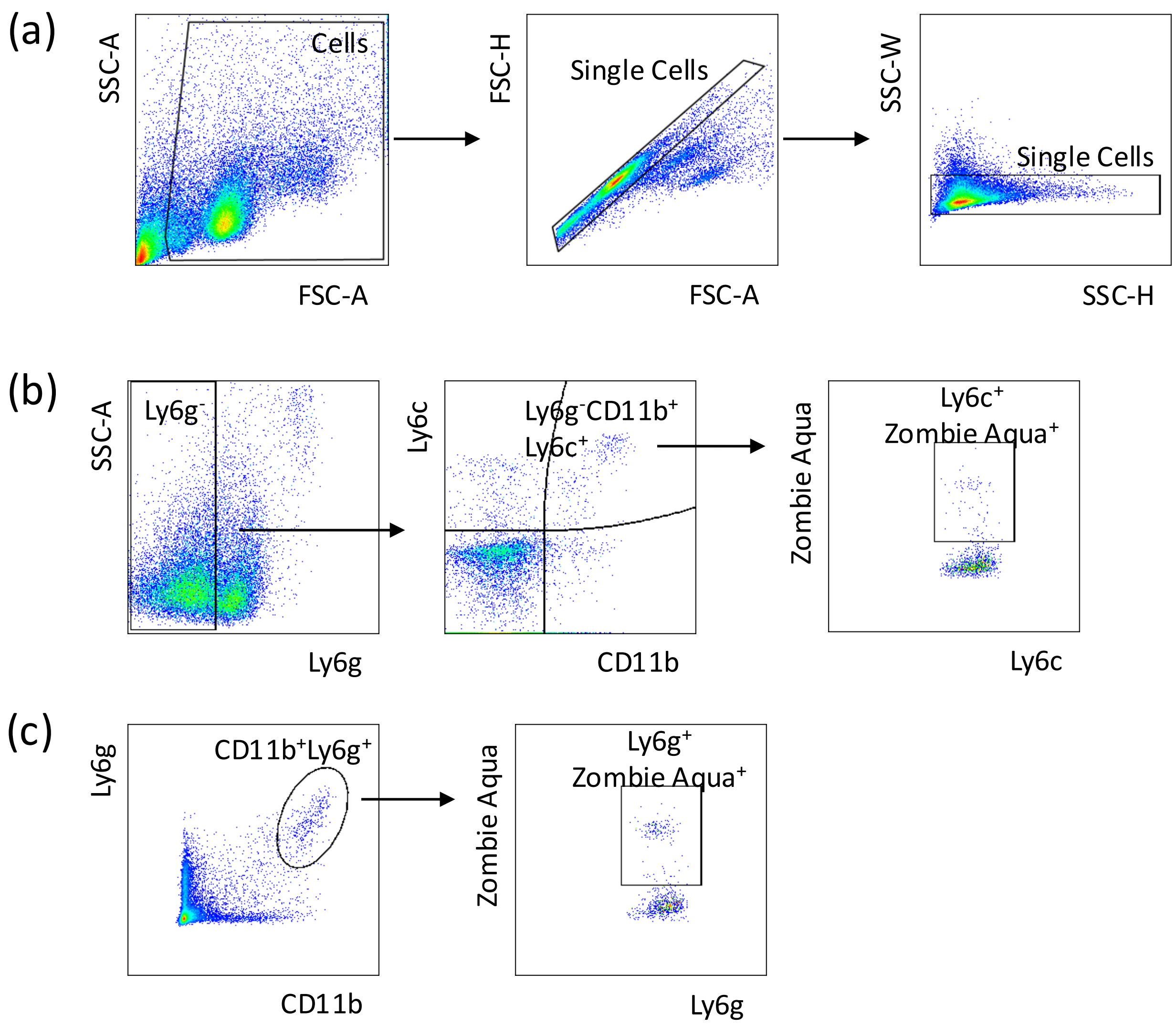

(d)

Cell Type	Cell Surface Marker
Monocytes	Ly6g$^-$CD11b$^+$Ly6c$^+$
Neutrophils	CD11b$^+$Ly6g$^+$

Supp. Figure 1. Gating strategy for defining effector cell subpopulations by flow cytometry. Briefly, doublets were excluded using Forward Scatter (FSC) and Side Scatter (SSC) as shown in **(a)** before gating for specific effector populations based on CD11b versus **(b)** Ly6c or **(c)** Ly6g expression.

Supp Figure 2

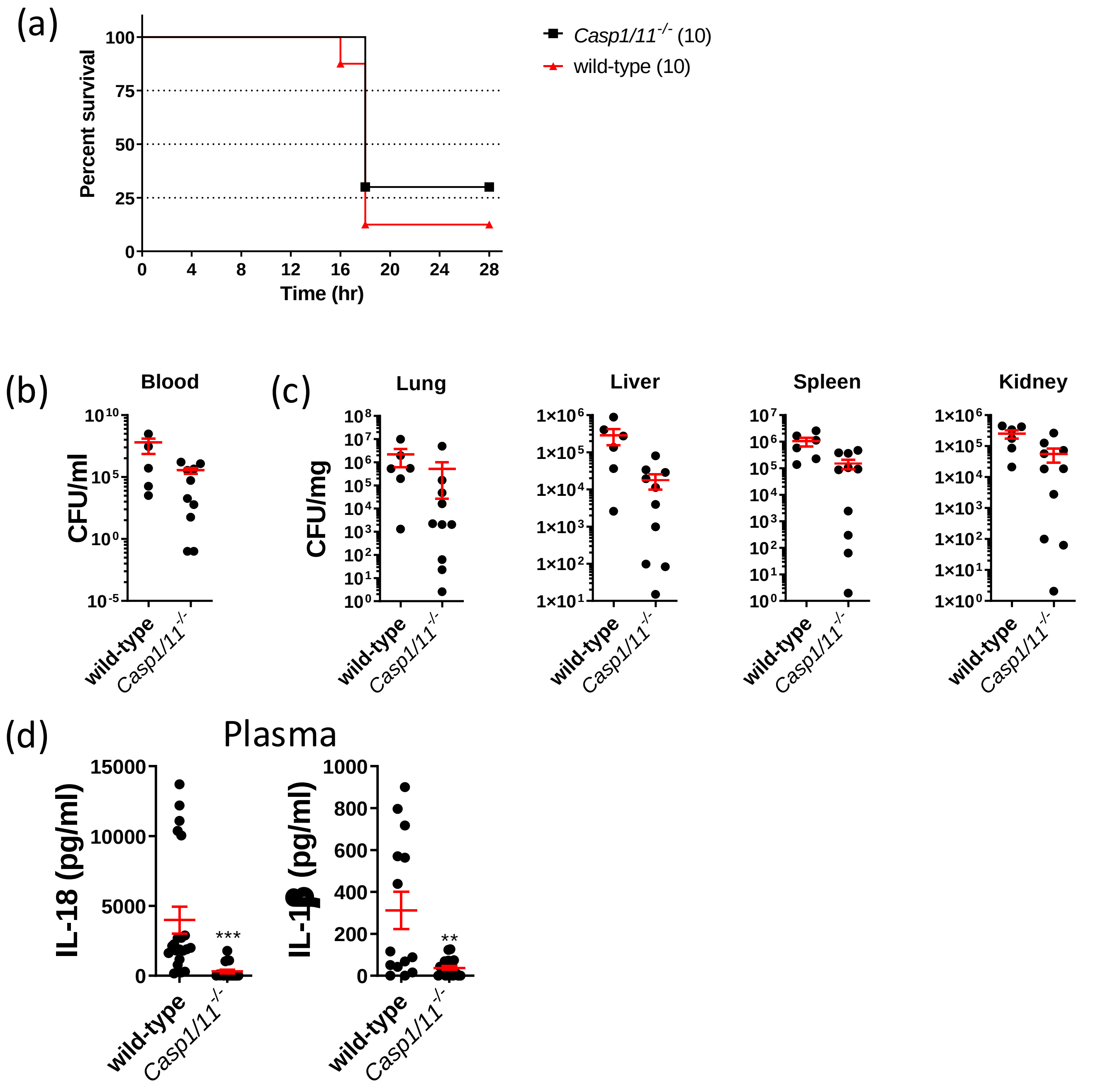

Suppl. Figure 2. *Caspase1/11$^{-/-}$* mice are susceptible to *A. baumannii* infection. (a) *Caspase1/11$^{-/-}$* mice survival rate, **(b)** the level of bacteriemia, **(c)** bacteria dissemination to different organ, and **(d)** plasma cytokine levels 16-20 hours post *A. baumannii* 1605 infection (i.p. 2×10^7 CFU/mouse). Data were collected from at least three independent experiments, n as indicated in parentheses, **, P < 0.01, ***, P < 0.001 compared to wild-type. mean ± SEM. Kaplan-Meier estimate was used to compare mice survival rates. Non-parametric t-test was used to compare differences between groups.

Supp Figure 3

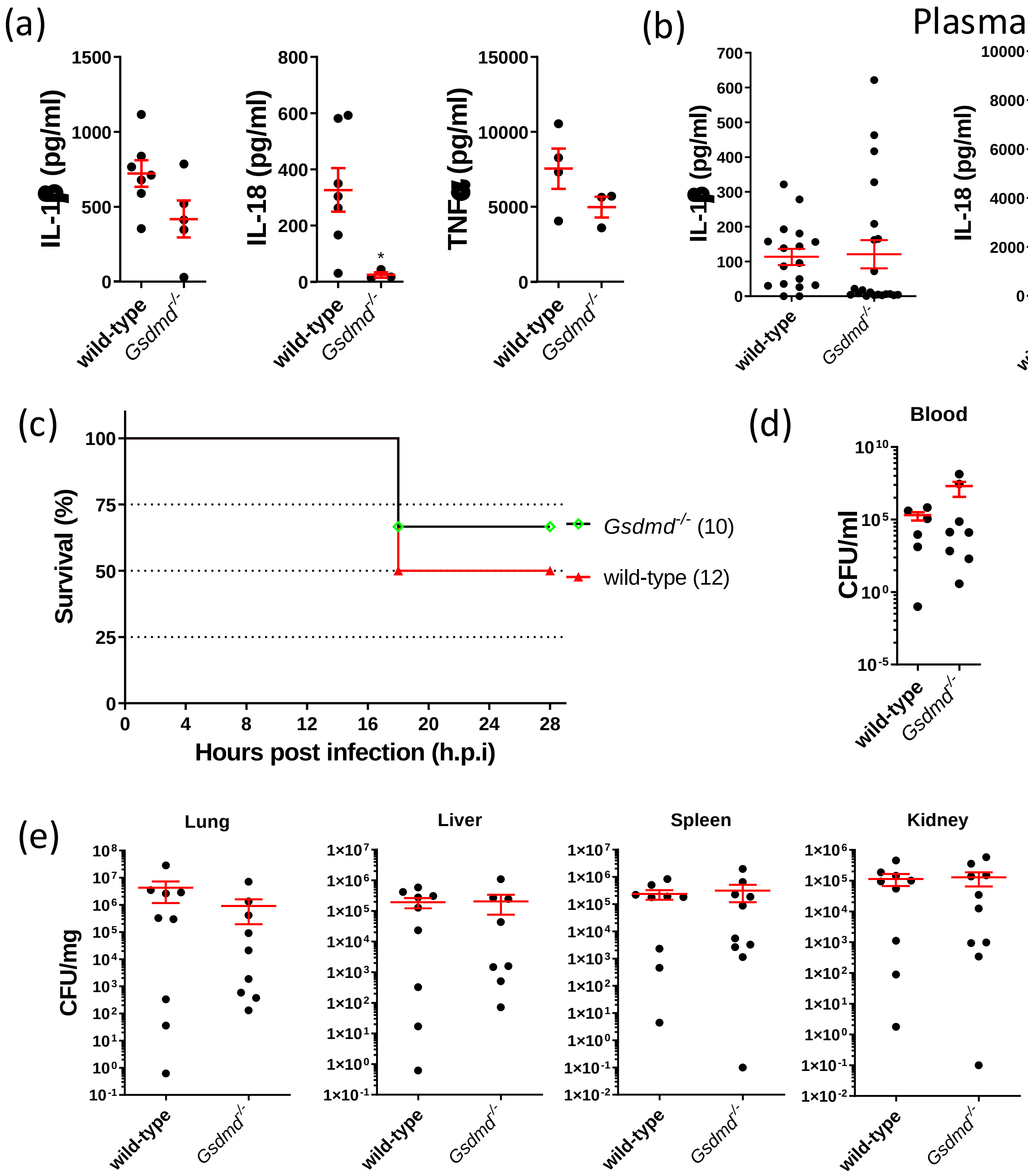

Suppl. Figure 3. Deleterious inflammation drives acute lethality in *A. baumannii*-infected mice. (a) BMDM cytokine levels IL-1β, IL-18 and TNFα in supernatants post 12 hours *A. baumannii* infection (m.o.i.=10), n = 5. **(b)** Mouse cytokine levels, **(c)** survival rate, **(d)** the level of bacteriemia and **(e)** bacteria dissemination to different organs 16-20 hours post *A. baumannii* 1605 infection (i.p. $2x10^7$ CFU/mouse). Data were collected from at least three independent experiments, n as indicated in parentheses, *, P < 0.05, **, P < 0.01, ***, P < 0.001 compared to wild-type. mean ± SEM. Kaplan-Meier estimate was used to compare mice survival rates. Non-parametric t-test was used to compare differences between groups.

Supp Figure 4

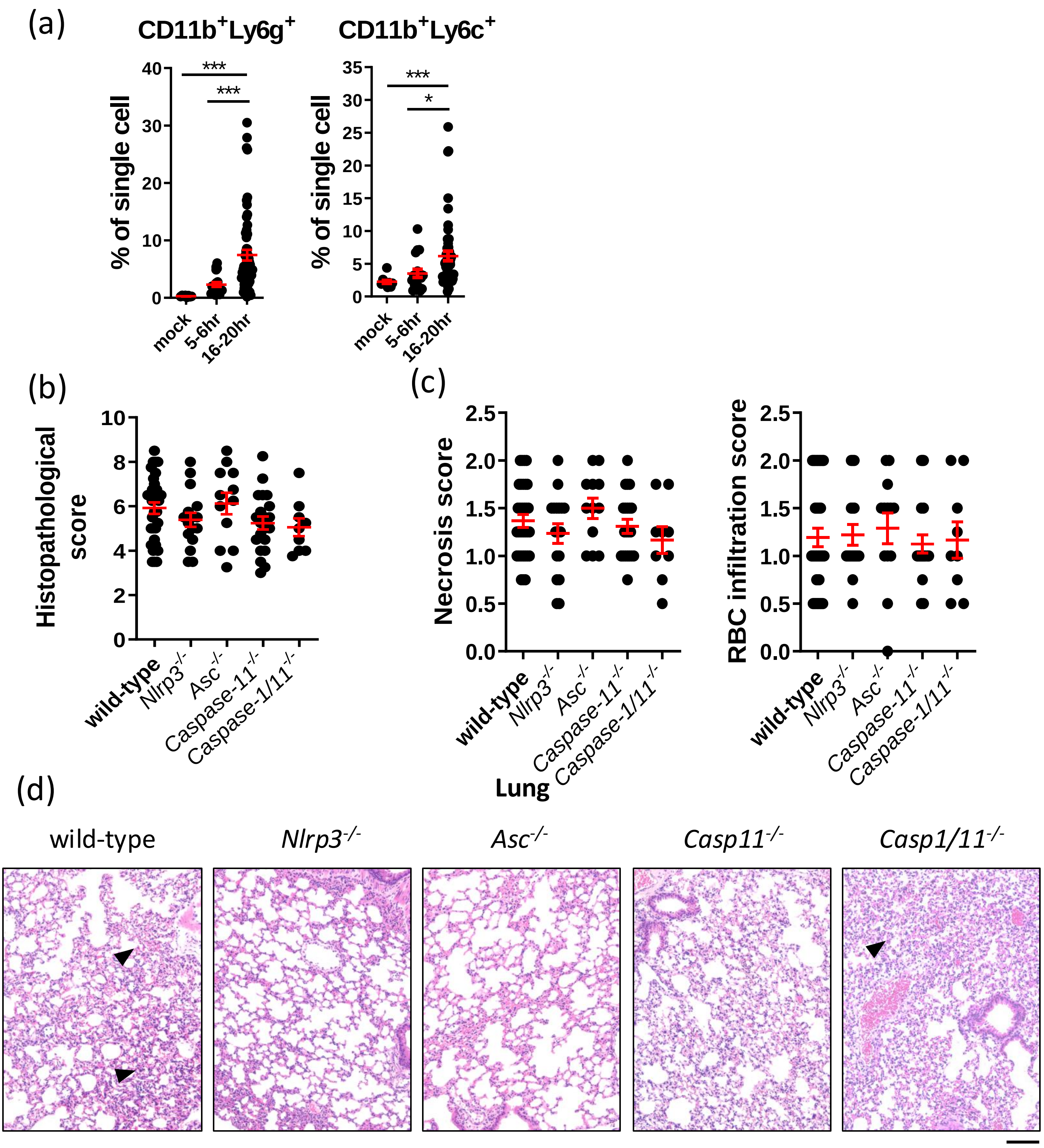

Supp. Figure 4. Recruitment of effector cells does not contribute to lung lesions. (a) Recruitment of neutrophils and inflammatory monocytes to the lung, **(b)** Lung histological scores, **(c)** necrosis or red blood cell (RBC) infiltration score and the **(d)** representative H&E staining of infected C57BL/6 mice 16-20 hours post *A. baumannii* 1605 infection (i.p. $2x10^7$ CFU/mouse). Arrowhead: immune cell infiltrate. n10-20 n=6-12, each data point represents a replicate. For each group, scale bar: 20 µm. Non-parametric t-test was used to compare differences between groups.

Supp Figure 5

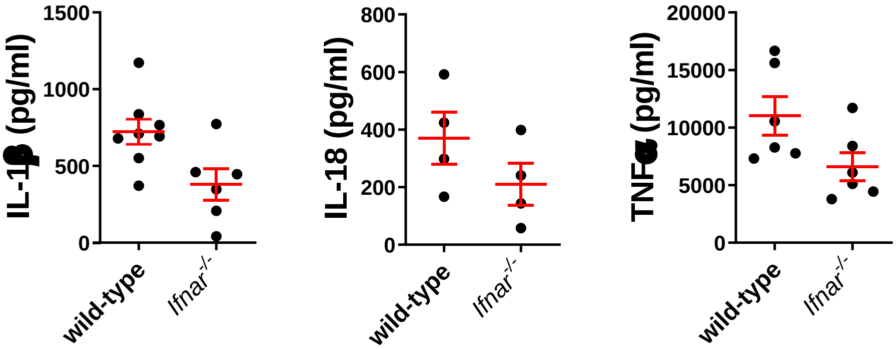

Supp. Figure 5. Absence of type I IFN signalling does not alter cytokine levels. Levels of cytokines IL-1β, IL-18 and TNFα in BMDM supernatants 12 hours post *A. baumannii* infection (m.o.i. 10), n = 4-8, each data point represents a replicate, mean ± SEM.

Supp Figure 6

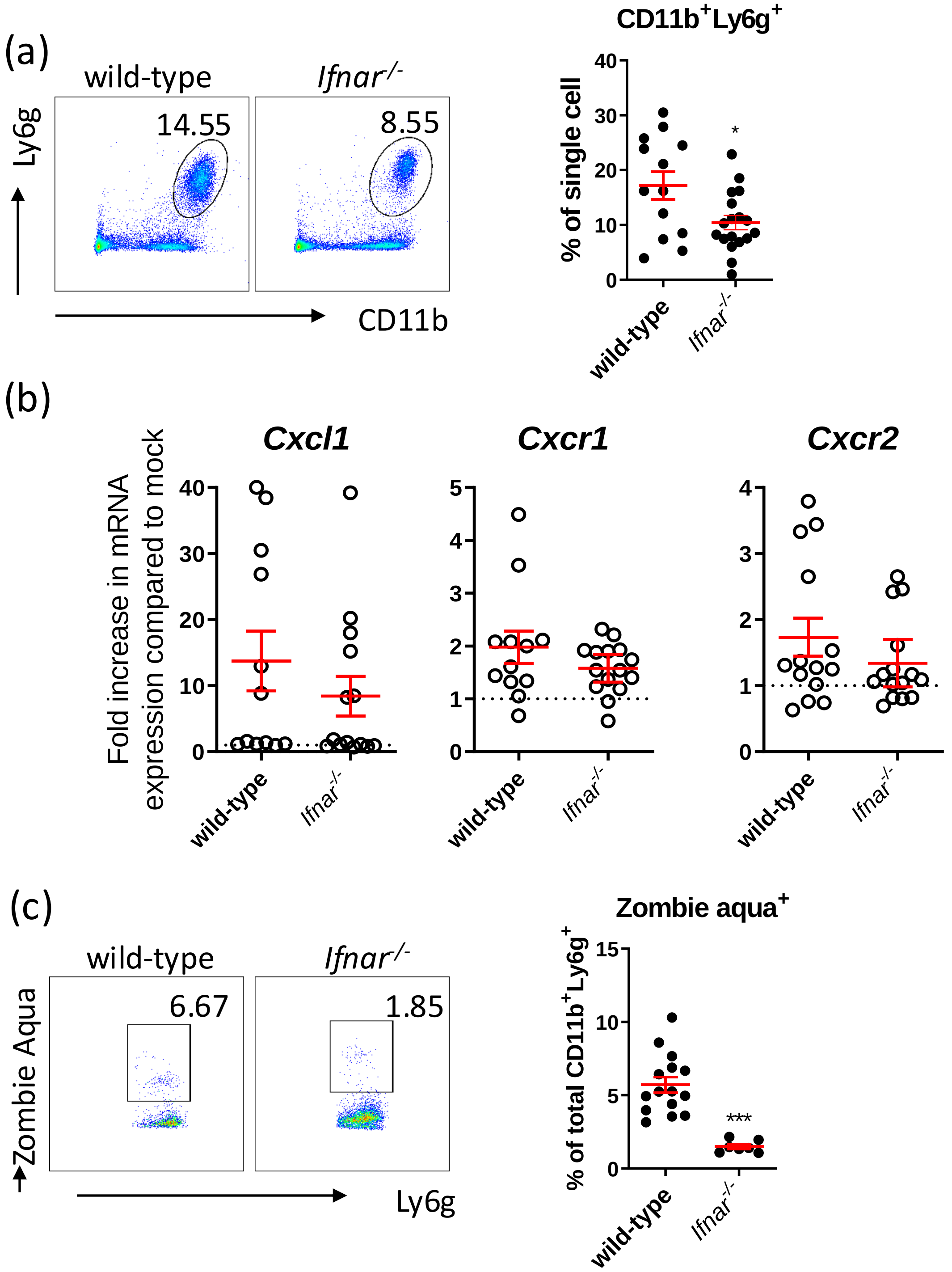

Supp. Figure 6. Absence of type I IFN signalling decreases neutrophil recruitment and neutrophil death. Flow cytometry quantification of **(a)** neutrophils ($CD11b^{+}Ly6g^{+}$) **(b)** qPCR quantification of induction of neutrophil chemokine and chemokine receptors **(c)** neutrophil cell death, in mice lung 14-20 hours post *A. baumannii* 1605 infection (i.p. $2x10^{7}$ CFU/mouse). Data were collected from at least three independent experiments. *, P < .05, ***, P < .001, mean ± SEM, n = 10-17, each data point represents a replicate. Non-parametric t-test was used to compare differences between groups.

Supp Figure 7

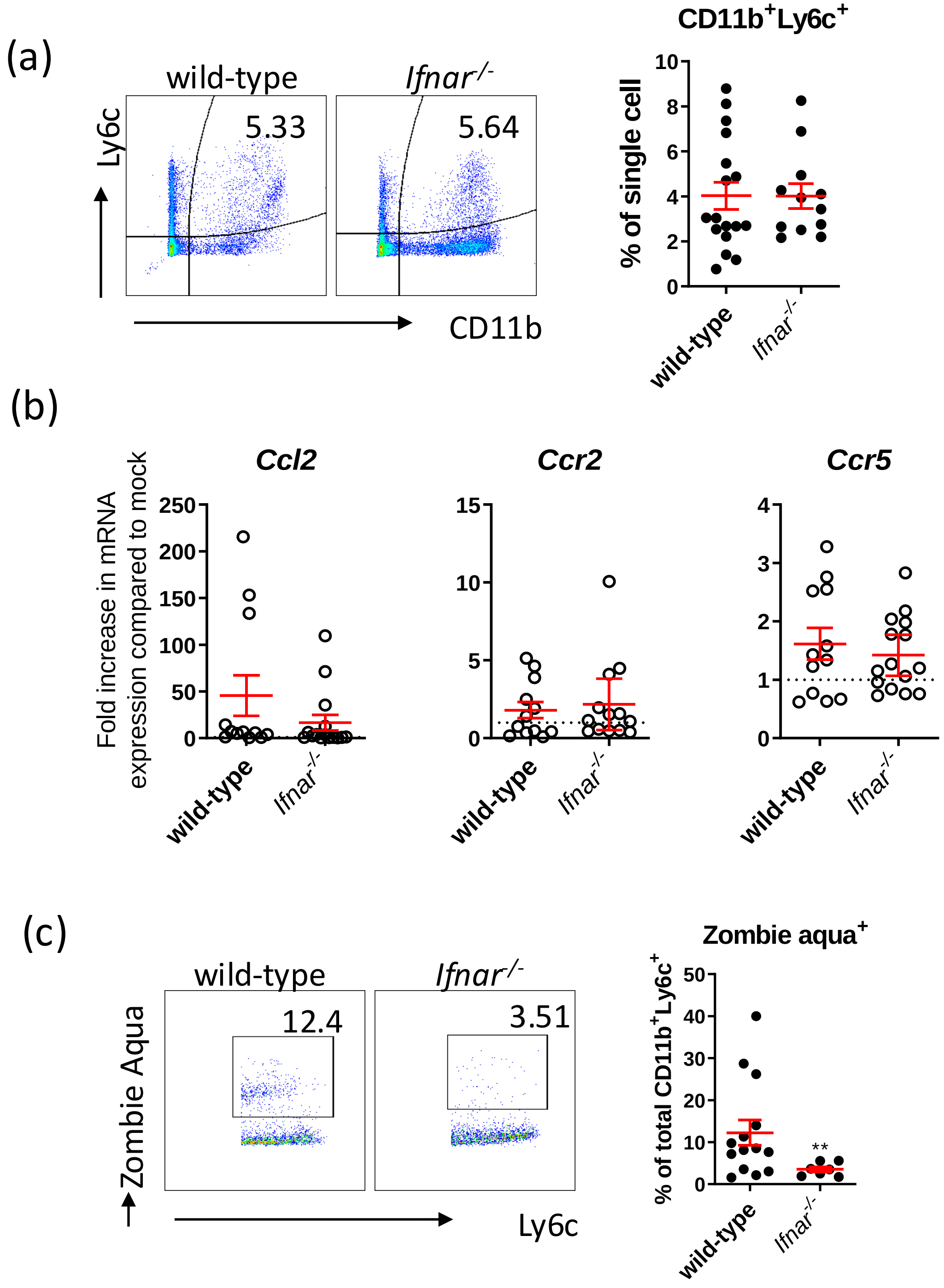

Supp. Figure 7. Absence of inflammasome signalling decreases inflammatory monocyte death only. Flow cytometry quantification of **(a)** inflammatory monocytes ($CD11b^{+}Ly6c^{+}$) **(b)** qPCR quantification of induction of inflammatory monocytes chemokine and chemokine receptors **(c)** Inflammatory monocytes cell death, in mice lung post 14-20 hours of *A. baumannii* 1605 infection (i.p. $2x10^{7}$ CFU/mouse). Data were collected from at least three independent experiments n = 12-17 for each group, n=6-12, each data point represents a replicate. **, P < 0.01, mean ± SEM. Non-parametric t-test was used to compare differences between groups.

Supp Figure 8

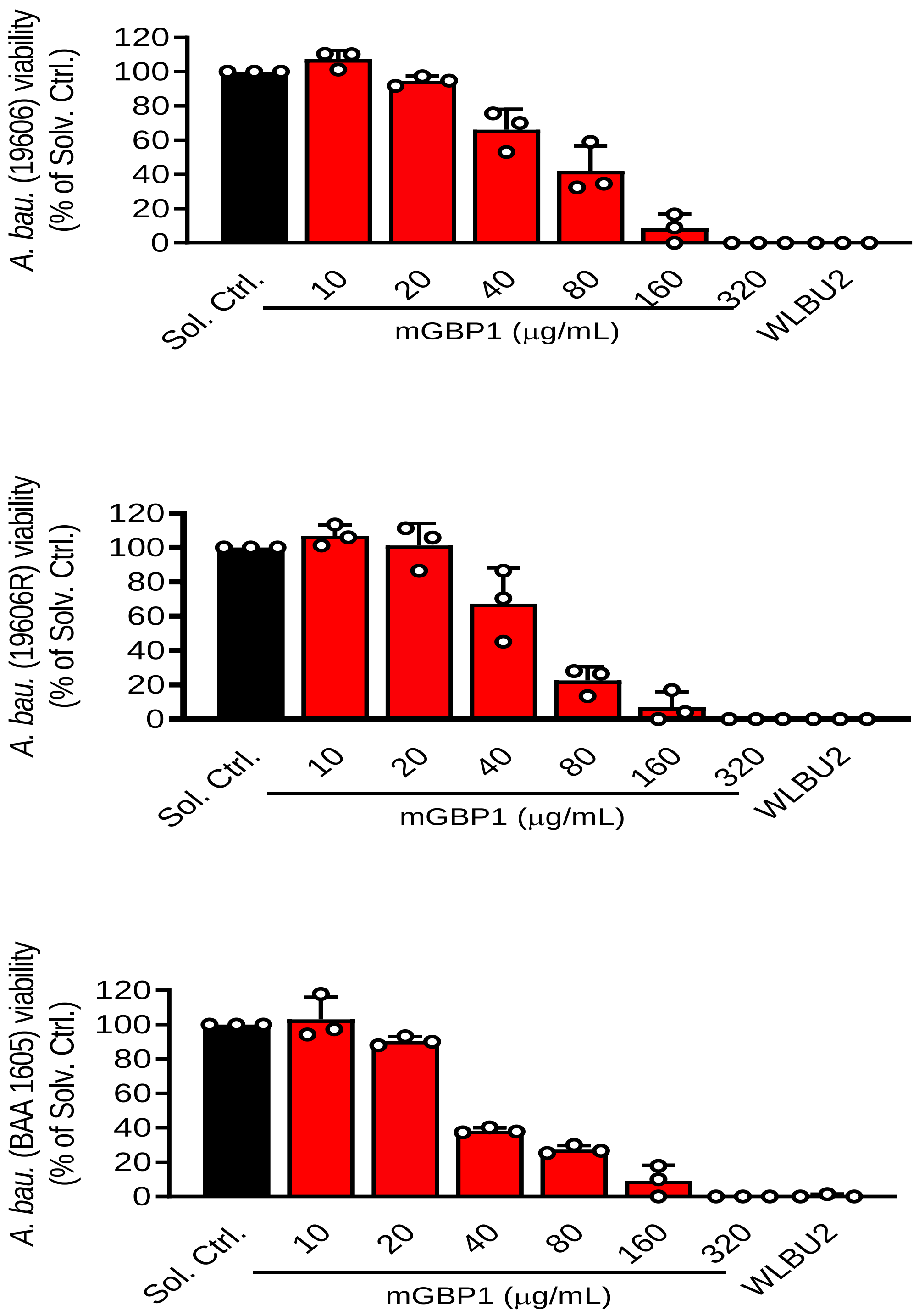

Supp. Figure 8. ***A. baumannii*** **viability post incubation with full-length mouse GBP1 protein.** Co-incubation of the full length purified GBP1 human protein at 10,20,40,80,160 and 320 µg/ml concentration with *A. baumanii* bacteria. CFU were measured 6 hours post incubation. Viability was measured as the CFU count relative to negative control and plotted as a percentage of viable cells. *A. baumannii* 1606, 19606 and 19606R were assessed whereas AL1847 could not. No statistical difference was found after multiple testing. N=3 independent experiments.

Supp Figure 9

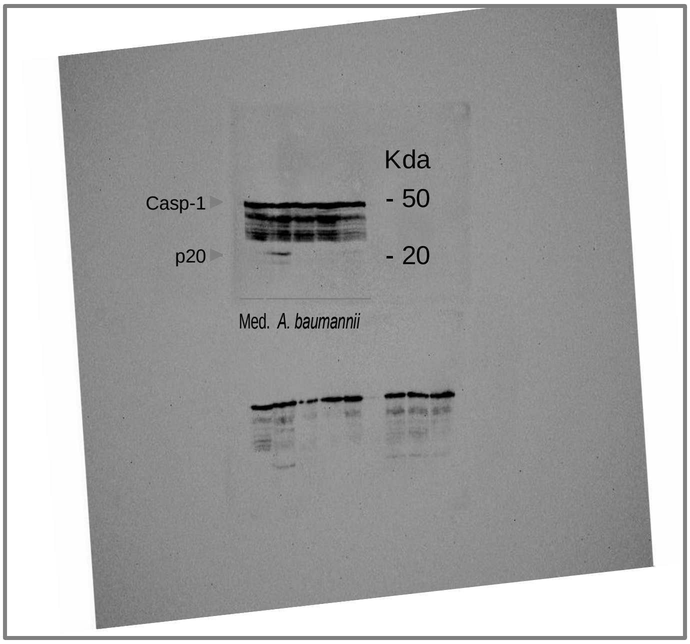

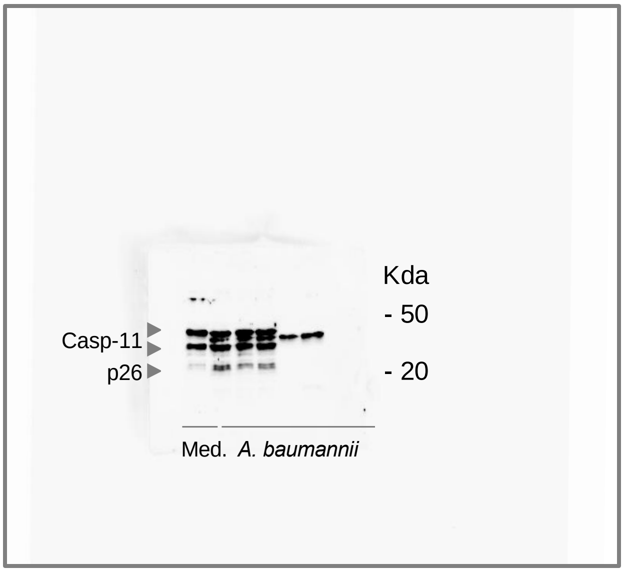

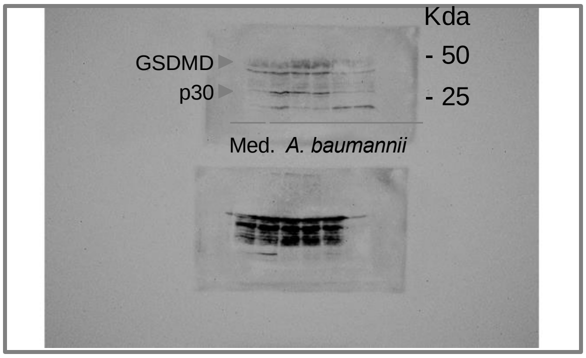

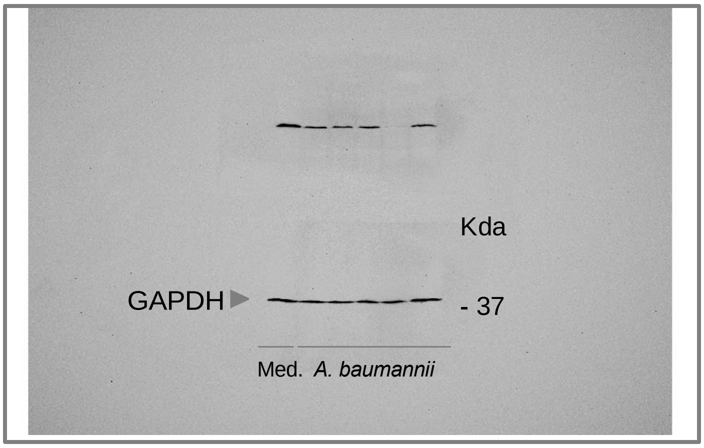

Supp. Figure 9. Uncropped Western Blot from Figure 1c

Supp Figure 10

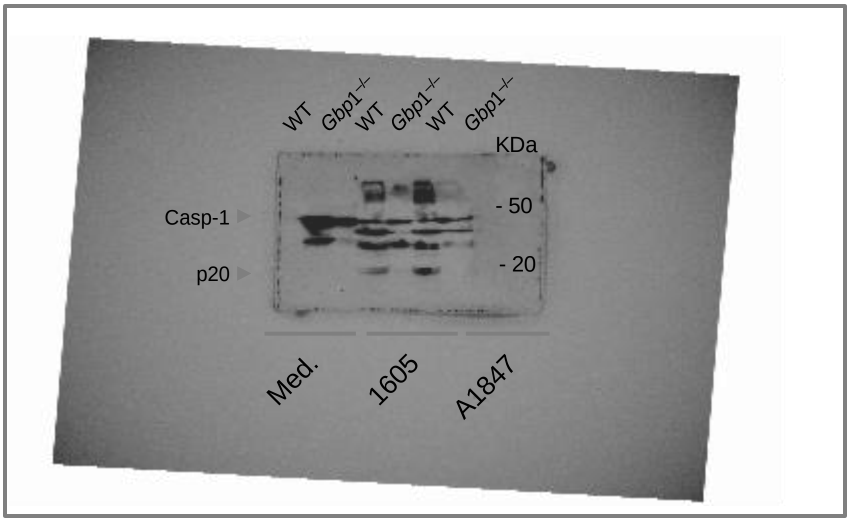

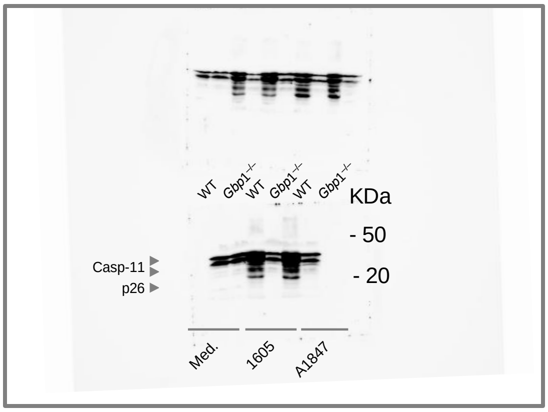

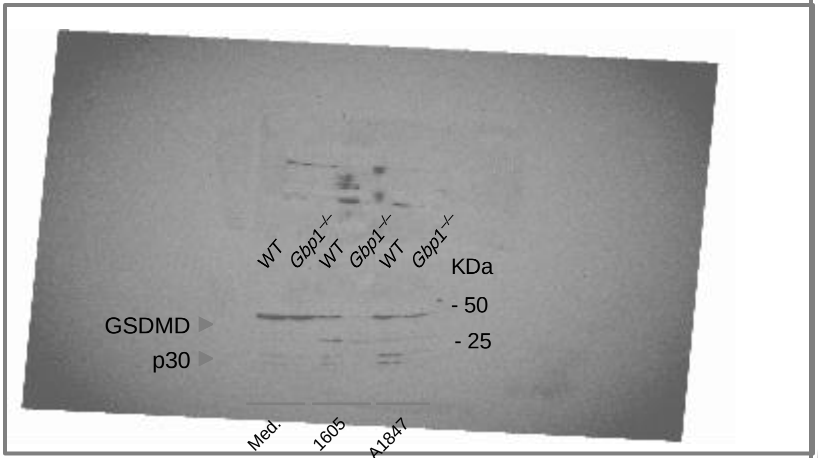

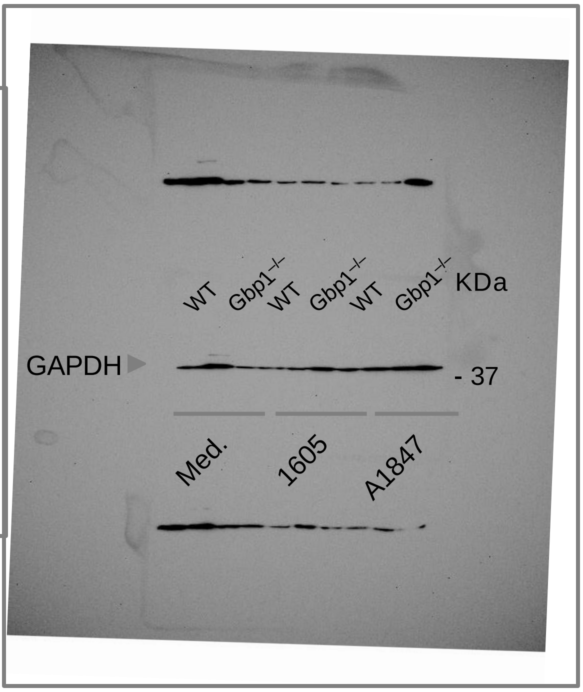

Supp. Figure 10. Uncropped Western Blot from Figure 7a

•**Supplementary Tables:**

Supplementary Table 1. qPCR primers used in this study

Gene	Forward	Reverse
**Nlrc4**	cta cat tga tgc tgc ctt gg	tct ctt cgt ctc tga gtc tc
**Aim2**	gat tca aag tgc agg tgc gg	tct gag gct tag ctt gag gac
**Nlrp3**	gtg gtg acc ctc tgt gag gt	tct tcc tgg agc gct tct aa
**Caspase-11**	aca atg ctg aac gca gtg ac	ctg gtt cct cca ttt cca ga
**Cxcl1**	gct tga agg tgt tgc cct cag	aag cct cgc gac cat tct tg
**Cxcr1**	aac ttt ggc att gtg gaa gg	cag cag cag gat acc act ga
**Cxcr2**	aac ttt ggc att gtg gaa gg	cga ggt gct agg att tga gc
**Ccl2**	gca tcc acg tgt tgg ctc a	ctc cag cct act cat tgg gat ca
**Ccr2**	aac ttt ggc att gtg gaa gg	gga aag agg cag ttg caa ag
**Ccr5**	aac ttt ggc att gtg gaa gg	ttc cta ctc cca agc tgc at
**Gapdh**	gag gaa cct gcc aag tat g	tgg gag ttg ctg ttg aag
