# Supplementary material for: Interferon signalling and non-canonical inflammasome activation promote host protection against multidrug-resistant Acinetobacter baumannii
Source: Commun Biol. 2024 Nov 12;7:1494. doi: 10.1038/s42003-024-07204-3 (PMC11557958; doi:10.1038/s42003-024-07204-3)
Supplement: Supplementary file 3 — Description of Additional Supplementary File [file 42003_2024_7204_MOESM3_ESM.pdf]

Description Of Additional Supplementary File

File name: Supplementary Data 1

Description: Numerical source data for graphs and charts
